# Left atrial and pulmonary venous remodeling following sutureless repair of pulmonary vein obstruction after catheter ablation

**DOI:** 10.1186/s13019-026-04013-z

**Published:** 2026-04-15

**Authors:** Yusuke Sakurai, Hirotaka Watanuki, Masato Tochi, Kayo Sugiyama, Katsuhiko Matsuyama

**Affiliations:** Department of cardiac surgery, Department of thoracic surgery, Aichi Medical University, Aichi, Japan

**Keywords:** Catheter ablation, Pulmonary vein obstruction, Sutureless repair

## Abstract

**Background:**

Severe pulmonary vein obstruction (PVO) is a rare but potentially serious complication following catheter ablation. In recent years, sutureless repair of PVO has been reported, with early.

**Methods:**

We performed sutureless repair on eight cases of symptomatic PVO and evaluated the mid-term surgical outcomes. All patients were diagnosed with right or left pulmonary vein obstruction using computed tomography, and complete loss of pulmonary blood flow in the affected area was confirmed by pulmonary perfusion scintigraphy.

**Results:**

Following surgery, symptoms resolved in all cases. Pulmonary perfusion scintigraphy was performed in all cases, confirming a significant improvement in pulmonary blood flow at the lesion sites. The atrial space observed immediately after surgery tended to decrease over the 6 to 12 months postoperatively, with the left atrium showing signs of remodeling during this period.

**Conclusions:**

Our sutureless repair technique has demonstrated favorable mid-term outcomes and may serve as a viable alternative to traditional surgical. methods.

## Background

Although catheter ablation for atrial arrhythmias has increased in recent years, severe pulmonary vein stenosis (PVS) or obstruction (PVO) remains a rare but potentially serious complication following the procedure. The incidence rate is reported to be approximately 1% [1]. PVO or PVS is associated with symptoms such as fever, hemoptysis, and chest pain. If left untreated, lung function in the affected area deteriorates, eventually necessitating consideration of pulmonary resection [2]. Although catheter intervention is commonly used as the first-line treatment for PVS, high restenosis rates persist, and mid-term outcomes remain unknown [3]. Additionally, completely occluded lesions cannot be accessed through catheter intervention. On the other hand, although surgical repair techniques such as direct repair or patch plasty are employed, restenosis remains a problem during follow-up. Recently, sutureless repair of PVO has been reported, showing early favorable results [4]. Nevertheless, there are no reports on long-term outcomes. In this report, we describe our experience with several cases and discuss their mid-term results.

## Patients and methods

This study included eight consecutive patients who underwent a modified sutureless technique for symptomatic PVO at Aichi Medical University Hospital (Aichi, Japan) between March 2022 and June 2025. All patients were diagnosed with right or left pulmonary vein obstruction using computed tomography, and complete loss of pulmonary blood flow in the affected area was confirmed by pulmonary perfusion scintigraphy. Due to the complete obstruction of the pulmonary vein ostia, radiologists determined that catheter-based treatment was not feasible. Therefore, no attempts were made to pursue this approach.

The surgical procedure, as described in a previously published paper, employed a sutureless technique using the left atrial appendage and a bovine pericardial patch [4]. However, in case 3 and subsequent cases, patch closure was omitted in the dissected extra-pericardial area surrounding the pulmonary vein and its branches. The key steps (namely, exposure of the pulmonary veins, incision lines, management of venous confluence and branches, pericardial patch suture lines, and patch geometry) were identical in all cases. The rationale for the modification was to reduce operative time, with the associated risk being thromboembolism. If atrial fibrillation recurred preoperatively, the Maze procedure was also performed simultaneously. Postoperative multidetector cardiac computed tomography (MDCT) was performed at 1 week, 6 months, 1 year, and 2 years to observe the morphology of the pulmonary veins and left atrium.

## Results

**Fig. 1:**
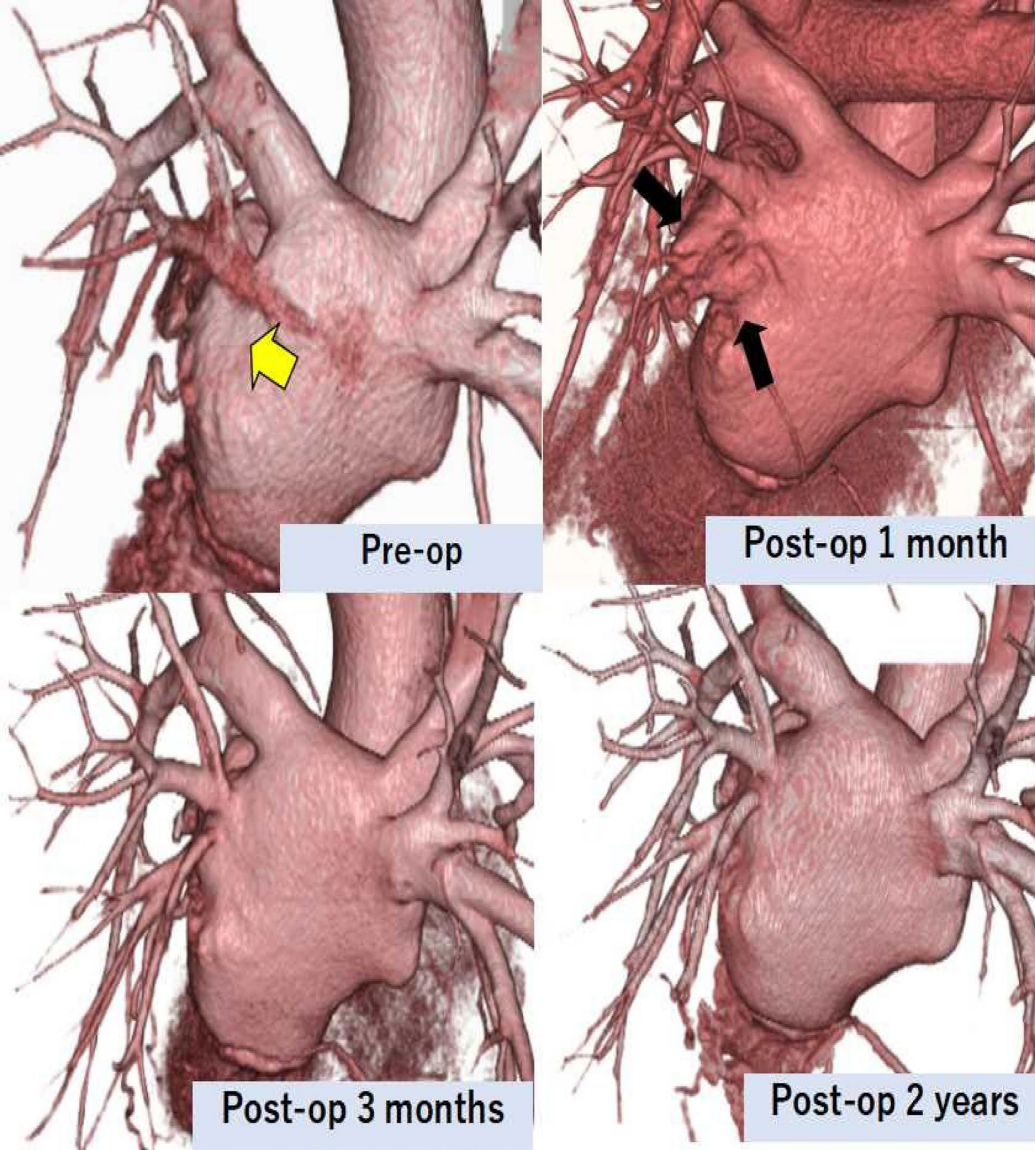
Three-dimensional cardiac computed tomography image (Case 2) : The yellow arrow indicates the site of pulmonary vein obstruction. An additional space (black arrows) involving the left atrial appendage was observed in the left atrium during the early postoperative period. However, the atrial space observed immediately after surgery tended to decrease over the three months postoperatively, with the left atrium showing signs of remodeling during this time. Pulmonary vein branches were observed without any obstruction

**Fig. 2:**
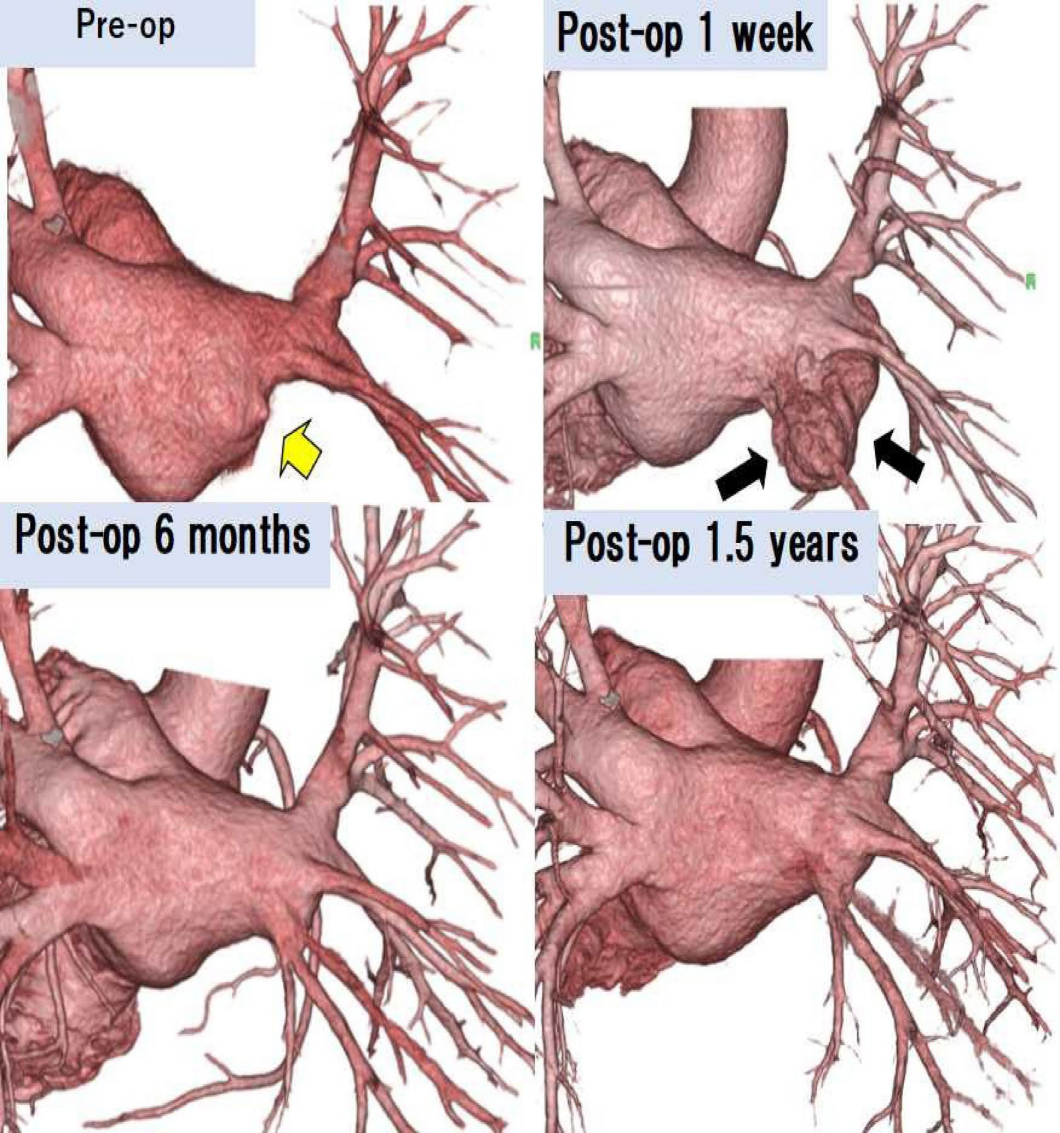
Three-dimensional cardiac computed tomography image (Case 4) : The yellow arrow indicates the site of pulmonary vein obstruction. An additional space (black arrows) involving the left atrial appendage was observed in the left atrium during the early postoperative period. Over time, the pulmonary vein appeared to form a direct anastomosis with the left atrium and gradually enlarged

Details of the cases are presented in Tables 1 and 2. In all eight cases, catheter ablation was performed to treat chronic atrial fibrillation. There were six males and two females. The ages ranged from 23 to 70 years, with a mean of 52.1 ± 18.7 years. Symptoms appeared within 3 to 9 months following ablation. The interval between ablation and surgery ranged from 4 to 19 months, with a mean of 9.9 ± 5.3 months. Following surgery, symptoms resolved in all cases. Pulmonary perfusion scintigraphy was performed in all cases, confirming a significant improvement in pulmonary blood flow at the lesion sites. In all five cases where the maze procedure was performed, sinus rhythm was successfully restored. Anticoagulation therapy was initiated with heparin during the acute postoperative period, followed by a transition to direct oral anticoagulants (DOAC). Concomitant antiplatelet therapy was not administered. After discharge, DOAC therapy was continued without interruption throughout the observation period, which ranged from 6 to 43 months, with a mean duration of 23 months. During the observation period, there were no cases of cerebral infarction or symptom recurrence. On MDCT, an additional space involving the left atrial appendage was observed in the left atrium during the early postoperative period (Fig. 1). However, the atrial space observed immediately after surgery tended to decrease over the three months postoperatively, with the left atrium showing signs of remodeling during this period. Moreover, the pulmonary vein gradually assumed a shape resembling a direct anastomosis to the left atrium over time, and the vein itself exhibited a progressive tendency to enlarge (Fig. 2).

**Table 1:** Symptoms and interval between ablation and symptom onset

	Age	Sex	BSA (m^2^)	Number of ablation	Symptoms	Interval 1 (months)
Case 1	51	m	1.59	1	Chest pain, Hemoptysis	3
Case 2	65	f	1.43	1	Chest pain, dyspnea	3
Case 3	24	m	1.61	1	Chest pain	5
Case 4	55	m	1.71	1	Chest pain, dyspnea, hemoptysis	9
Case 5	62	m	1.83	1	Dyspnea, hemoptysis	6
Case 6	23	m	1.67	1	Dyspnea, hemoptysis	3
Case 7	67	f	1.44	1	Dyspnea, hemoptysis	3
Case 8	70	m	1.75	2	Dyspnea, hemoptysis	6

BSA: Body surface area, Interval 1: interval between ablation and symptom onset

**Table 2:** PVO lesion and other characteristics

	PV lesion	Rhythm (pre-surgery)	Maze	Interval 2 (months)	Follow-up (months)
Case 1	LUL(S), LLL(CO)	Af	(+)	14	43
Case 2	LLL(CO)	SR		14	37
Case 3	LUL(SO), LLL(CO)	Af	(+)	7	36
Case 4	RLL(CO)	SR		19	25
Case 5	LUL(SO), LLL(CO)	Af	(+)	9	21
Case 6	LUL(SO), LLL(CO)	Af	(+)	4	11
Case 7	LUL(S), LLL(CO)	SR		4	11
Case 8	RUL(CO)	Af	(+)	8	6

PV: pulmonary vein, Interval 2: interval between ablation and surgery. LUL: left upper lobe, LLL: left lower lobe, RUL: right upper lobe, RLL: right lower lobe, S: severe stenosis, CO: complex obstruction, SO:simple obstruction, SR: sinus rhythm, Af: atrial fibrillation

## Discussion

Although the reported prevalence of PVO following catheter ablation is approximately 1% [1], the incidence at our institution remains unknown because most of the eight cases were referred from other hospitals. Furthermore, since PVO is often undiagnosed in the absence of symptoms, the true prevalence may be higher.

The advantage of sutureless repair for acquired PVO unlike direct anastomosis, is that it prevents geometric distortion of the pulmonary venous structures, avoids trauma to the pulmonary venous wall and endothelium, and reduces the risk of subsequent intimal hypertrophy. Ultimately, the sutureless technique is expected to reduce the recurrence of PVO [5]. Although several case reports have described sutureless repair for PVO, no studies have reported mid-term outcomes. In this study, we performed sutureless repair on eight cases of PVO and evaluated the mid-term surgical outcomes.

Symptoms disappeared in all patients postoperatively, and no cases of recurrence were observed during the follow-up period. The results of the maze procedure performed at the same time were also favorable. Three-dimensional cardiac computed tomography revealed an enlarged space within the left atrium, including the left atrial appendage or the extra-pericardial sac, during the early postoperative period. However, 6 to 12 months later, this space had resolved, and remodeling of the left atrium and pulmonary vein branches was observed without obstruction of the pulmonary vein ostium. The pulmonary vein assumed a shape that appeared to fit directly into the left atrium and exhibited a tendency to gradually expand. However, a limitation of this paper is that it evaluates remodeling using MDCT only qualitatively. Although quantitative evaluation is preferable, it is challenging to perform this assessment from the images.

The occlusion in pediatric PVO is limited to the central portion only. In contrast, adult PVO following ablation involves a longer and more complex occluded segment, often necessitating dissection extending into the peripheral branches.Therefore, thrombus formation may occur in the extra-pericardial area created by the extended dissection.

Anticoagulation therapy with heparin or DOAC was initiated early postoperatively. Fortunately, none of the patients experienced complications related to cerebral infarction. DOAC administration is currently ongoing in all cases, with no discontinuations reported to date. Concomitant antiplatelet therapy is not being administered. The optimal duration of DOAC therapy remains a subject of debate and warrants further research.

In conclusion, due to a shortage of data and insufficient time for post-operative follow-up, the long-term results remain uncertain. However, our sutureless repair has demonstrated favorable mid-term outcomes and may serve as a viable alternative to traditional surgical methods.

## Data Availability

The data underlying this article will be shared on reasonable request to the corresponding author.

## Abbreviations

PVS: Pulmonary vein stenosis
PVO: Pulmonary vein obstruction
MDCT: Multidetector cardiac computed tomography
DOAC: Direct oral anticoagulants

## References

[1] Teunissen C, Velthuis BK, Hassink RJ, van der Heijden JF, Vonken EPA, Clappers N, et al. Incidence of pulmonary vein stenosis after radiofrequency catheter ablation of atrial fibrillation. JACC Clin Electrophysiol. 2017;3:589–98. 29759432 10.1016/j.jacep.2017.02.003

[2] Papakonstantinou NA, Zisis C, Kouvidou C, Stratakos G. Lobectomy due to pulmonary vein occlusion after radiofrequency ablation for atrial fibrillation. Korean J Thorac Cardiovasc Surg. 2018;51:290–2. 30109211 10.5090/kjtcs.2018.51.4.290 PMC6089619

[3] Fender EA, Widmer RJ, Hodge DO, Packer DL, Holmes DR Jr. Assessment and management of pulmonary vein occlusion after atrial fibrillation ablation. JACC Cardiovasc Interv. 2018;11:1633–9. 10.1016/j.jcin.2018.05.020 30139471

[4] Matsuyama K, Watanuki H, Tochii M, Sugiyama K. A modified sutureless repair for left pulmonary vein obstruction after catheter ablation. Interact Cardiovasc Thorac Surg. 2022;35:ivac097. 35389491 10.1093/icvts/ivac097 PMC9297515

[5] Najm HK, Caldarone CA, Smallhorn J, Coles JG. A sutureless technique for the relief of pulmonary vein stenosis with the use of in situ pericardium. J Thorac Cardiovasc Surg. 1998;115:468–70. 9475545 10.1016/S0022-5223(98)70294-6

